# Indolizinoquinolinedione Metal Complexes: Structural Characterization, In Vitro Antibacterial, and In Silico Studies

**DOI:** 10.3390/molecules31020348

**Published:** 2026-01-19

**Authors:** Jacopo Vigna, Michael Marchesi, Ibtissem Djinni, Miša Mojca Cajnko, Kristina Sepčić, Andrea Defant, Ines Mancini

**Affiliations:** 1 Laboratory of Bioorganic Chemistry, Department of Physics, University of Trento, 38123 Trento, Italy; jacopo.vigna@unitn.it (J.V.); michael.marchesi@studenti.unitn.it (M.M.); 2 Laboratory of Applied Microbiology, Faculty of Natural and Life Sciences, University of Bejaia, Targa Ouzemmour, Béjaïa 06000, Algeria; ibtissem.djinni@yahoo.fr; 3 Department of Biology, Biotechnical Faculty, University of Ljubljana, Jamnikarjeva 101, 1000 Ljubljana, Slovenia; misa.cajnko@ki.si (M.M.C.); kristina.sepcic@bf.uni-lj.si (K.S.)

**Keywords:** Indolizinoquinoline-5,12-dione derivatives, metal chelates, Job’s plot, antibiotic resistance, MRSA, *Acinetobacter baumanii*, MIC evaluation, molecular docking, density functional theory (DFT) calculation

## Abstract

In the search for solutions to the global health threat posed by antimicrobial resistance, the development of new compounds is crucial. In this context, the in vitro testing of known indolizinoquinolinedione analogs **1**–**7** revealed that *N*,*N*-syn regioisomers are more active than *N*,*N*-anti regioisomers. In particular, compound **2** (ethyl 5,12-dihydro-5,12-dioxoindolizino[2,3-*g*]quinoline-6-carboxylate) exhibited the most significant activity against *Bacillus subtilis*, *B. cereus*, *Staphylococcus aureus*, and methicillin-resistant *S. aureus* (MRSA) bacteria. The reported increased bioactivity of metal complexes and their ability to overcome drug resistance through metal coordination have induced the study of new metal complexes of compound **2**. FT-IR spectroscopy combined with DFT-simulated spectra confirmed the C=O chelation in all Zn, Cu, and Mn complexes **8**–**10**. ESI-MS isotopic cluster analysis and UV-Vis-derived Job’s plot provided significant evidence for 1:1 chelation. Finally, ^1^H NMR data were correlated to the DFT-calculated charge distribution. Complexes **8**–**10** displayed similar activity against *B. subtilis*, although this was lower than that for **2**, and there were comparable effects with 2 and vancomycin antibiotic against *S. aureus*. FTsZ protein as a potential target of *B. subtilis* and DNA gyrase of *S. aureus* and MRSA were studied by docking calculations, revealing a good correlation with the in vitro results.

## 1. Introduction

According to the World Health Organization (WHO), antimicrobial resistance (AMR), which includes resistance to bacteria, viruses, fungi, and parasites, is a top global public health concern. It occurs when microorganisms no longer respond to antimicrobial medicines, causing them to become ineffective and making infections difficult or impossible to treat. This resistance process is natural and happens over time through genetic changes in pathogens, which can acquire adaptations for survival and resistance mechanisms. In particular, the misuse and overuse of available drugs to treat and prevent infections in humans, animals, and plants accelerate their emergence and spread [1]. ESKAPE pathogens are a group of six clinically significant disease-causing bacteria (*Enterococcus faecium*, *Staphylococcus aureus*, *Klebsiella pneumoniae*, *Acinetobacter baumannii*, *Pseudomonas aeruginosa*, and *Enterobacter* spp.). *Escherichia coli* is sometimes included in this group. These bacteria have developed multidrug resistance, evading commonly used antibiotics such as penicillin, vancomycin, carbapenems, and others. Furthermore, they are a major cause of nosocomial infections, responsible for millions of deaths. The WHO has classified them among its priority pathogens based on their infection severity, antibacterial resistance, and the limited availability of effective antibiotics. *P. aeruginosa* and *S. aureus* are categorized within the high-priority group [2]. *S. aureus* is a major human pathogen causing various infections and is associated with hospital complications and mortality. Methicillin-resistant *S. aureus* (MRSA) exhibits resistance to most β-lactams and is a highly problematic pathogen [3]. *A. baumannii* and *K. pneumoniae* are Gram-negative bacteria associated mainly with severe infections in immunocompromised patients in intensive care units. Alarmingly, a noticeable increase in the frequency of *A. baumanii* has been observed in recent years, with the species often developing resistance to the penicillinase-resistant beta-lactam oxacillin treatment [4].

The progress in research on finding solutions to this has not kept pace with the accelerated emergence of antibiotic resistance. However, the urgent priority of discovering novel effective antimicrobial agents has recently relied on several approaches. One of these is based on studies of metal-based antimicrobials that can eliminate multidrug-resistant bacteria [5]. Not all metals are intrinsically toxic, and there are successful examples of metal complexes and organometallic molecules being used in medicine [6]. Most antibacterial metal complexes are able to inhibit Gram-positive bacteria (*S. aureus* and MRSA). Instead, fewer examples have been reported in effective inhibition against Gram-negative bacteria, mainly due to the more favorable membrane morphology of Gram-positive strains, which allows for easier entry of the antibacterial agent. Consequently, effective metal complexes against Gram-negative bacteria are lacking [6].

Structurally, the increased biological activity of metal complexes can be explained by an increased delocalization of π electrons across the entire chelate ring. This feature increases the lipophilicity of the central ion and thereby enhances the complex’s passage through the lipid membranes and its penetration into cells. Additionally, given the greater stability of the chelate in solvents with a lower dielectric constant [7], even the use of complexes that are not exceptionally stable in an aqueous environment can improve the pharmaceutical profile of the ligand by obtaining complexes with enhanced solubility. Even when the metal chelate cannot increase activity compared to the free ligand, the metal coordination can still overcome drug resistance, as demonstrated in the case of quinolone antibacterials [5].

**Figure 1:**
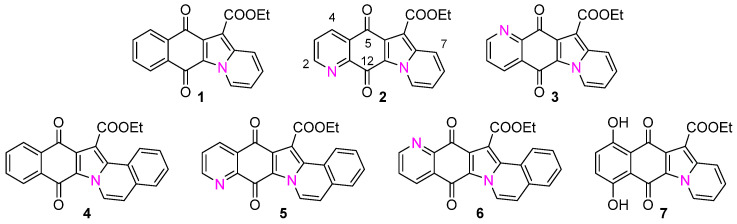
Molecular structures of indolizinoquinolinedione derivatives and analogs under investigation: ethyl 5,12-dihydro-5,12-dioxoindolizino[3,2-*g*]quinoline-11-carboxylate (**1**), ethyl 5,12-dihydro-5,12-dioxoindolizino[2,3-*g*]quinoline-6-carboxylate (**2**), ethyl 5,12-dihydro-5,12-dioxoindolizino[3,2-*g*]quinoline-11-carboxylate (**3**), ethyl 8,13-dihydro-8,13-dioxobenz[5,6]indolo[2,1-*a*]isoquinoline-14-carboxylate (**4**), ethyl 8,13-dihydro-8,13-dioxobenz[7,8]indolizino[2,3-*g*]quinoline-14-carboxylate (**5**), ethyl 8,13-dihydro-8,13-dioxobenz[7,8]indolizino[3,2-*g*]quinoline-14-carboxylate (**6**), ethyl 6,11-dihydro-7,10-dihydroxy-6,11-dioxobenzo[*f*]pyrido[1,2-*a*]indole-12-carboxylate (**7**).

In previous studies, we produced the indolizinoquinolinedione derivatives and analogs **1**–**7**. These were then further functionalized and tested for their ability to inhibit the growth of a panel of cancer cell lines [8]. This series included the benzopyridoindoledione **1**, its dihydroxy analog **7**, and the benzoindoloisoquinoline dione **4**, together with *N*,*N*-syn and *N*,*N*-anti regioisomers **2/3** and **5/6** (Figure 1) [8,9,10,11].

In this work, we focused on the potential antibacterial activity of analogs **2**–**7**, expanding the study of the chemical space of this heterocycle scaffold. A Chinese patent also reported the antimicrobial data of compound **2** and its related 7-OH and 7-NH_2_ methyl and ethyl esters analogs, identifying compound **2** as the most active one [12].

Additionally, we have synthesized some metal complexes with compound **2** as a ligand, paying particular attention to their structural characterization under different solvent conditions. Ligand **2** was selected because it was the most active in our evaluation of a series of Gram-positive and Gram-negative pathogenic bacteria. The observed antibacterial activities were also supported by docking calculations considering detailed targets for different bacterial strains.

## 2. Results and Discussion

### 2.1. Synthesis of Molecules **1**–**7**

**Scheme 1:**
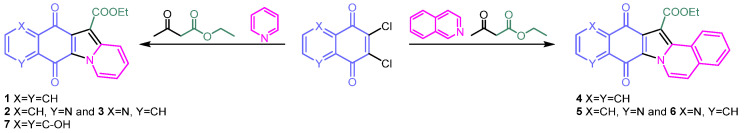
Synthetic procedure for obtaining molecules **1**–**7**.

In a one-pot multicomponent cyclization for obtaining the desired compounds, ethyl acetoacetate and pyridine were treated with 2,3-dichloronaphthalene-1,4-dione to provide compound **1**, and with 6,7-dichloroquinoline-5,8-dione to give a mixture of the *N*,*N*-syn isomer **2** and *N*,*N*-anti isomer **3** (Scheme 1). The selectivity of this reaction is affected by the choice of solvent and the presence of a suitable metal salt. Regioisomers **2** and **3** were purified by column chromatography, and their structures were established by extensive NMR analysis and comparison with a reference compound derived from decarboxylation [10]. This synthetic procedure became more efficient when microwave irradiation replaced conventional heating [11]. Similarly, compound **7** was obtained starting from the commercial 2,3-dichloro-5,8-dihydroxy-1,4-naphtoquinone [9]. The aza- and diaza-dibenzofluorenedione esters **4** and **5**/**6** were accessible by cyclization of ethyl acetoacetate and isoquinoline with the corresponding dichloroquinone, respectively (Scheme 1) [11].

### 2.2. Synthesis and Structural Characterization of Metal Complexes

**Scheme 2:**
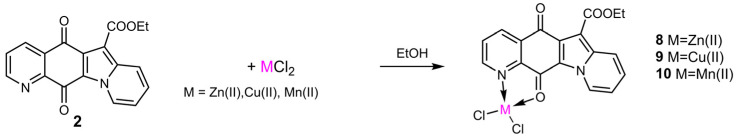
Synthesis of metal complexes **8**–**10**.

Compound **2**, the most active of analogs **1**–**7** in the antibacterial tests (see below), was considered as an *N*/*O* bidentate ligand for obtaining the metal complexes **8**–**10** (Scheme 2). The metal chelates were produced by treating compound **2** with the chloride salts of zinc, copper, and manganese divalent ions. We had already investigated a similar *N*,*O* metal chelation of 6,7-dichloroquinoline-5,8-dione, which was able to affect the regioselectivity of amination [10], where Raman spectroscopy combined with DFT calculations in the characterization of the key intermediate metal complexes could be used to detect the best selectivity [13].

The ability of metal ions to bind a ligand can be qualitatively predicted by Pearson’s acid–base theory, which provides an ordering of transition metals according to their preferences for specific organic ligands. In detail, Zn(II) and Cu(II) ions are acids with intermediate properties between hard and soft; they are therefore classified as borderline and have affinity with borderline bases, including aromatic nitrogen centers [5]. It is, for example, the case of the nitrogen present in the quinolinedione moiety of ligand **2**. Indeed, no complex was produced by treating analog **1** with zinc chloride.

Metal complexes **8**–**10** were obtained by reacting an equimolar amount of compound **2** with the suitable chloride salt in ethanol and evaporation of the solvent (Scheme 2).

Among metal-based complexes as promising antimicrobial agents, the choice of zinc chelation is based on the property of zinc ions to be nutrients for bacteria and on the report of active complexes, especially against *S. aureus*. In addition, Zn(II) complexes generally display lower toxicity, resulting in fewer side effects than other metal-based drugs [14].

**Figure 2:**
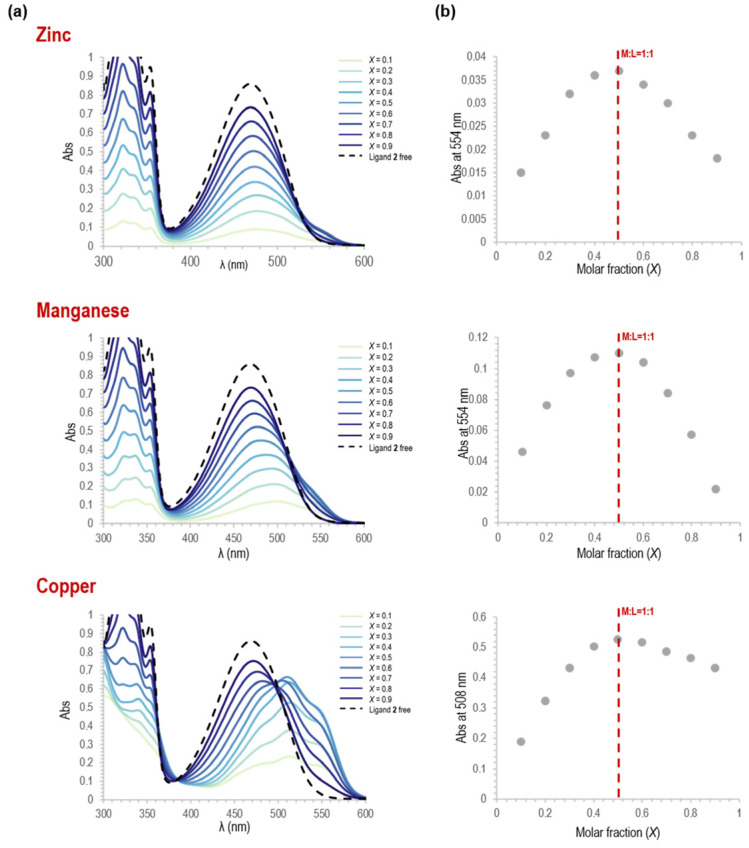
(**a**) Changes in absorbance during complex formation in acetonitrile starting from the solutions of MCl_2_ (with M=Zn, Cu, or Mn) or/and the ligand, both at 1.5 × 10^−4^ M, prepared in acetonitrile and then mixed in different molar ratios; (**b**) the corresponding Job’s plot: absorbance values as a function of the molar fraction *Χ* of the ligand **2** during the formation of the complex by adding metal chloride salts in acetonitrile at the indicated λ_abs_ values.

The structural characterization has been mainly studied in solution, considering also solvents that can reproduce the conditions used in the biological evaluation. We have investigated the UV absorption of **2** as a ligand in a Zn(II) complex solubilized in solvents of different polarity, whose choice was forced by having the chloride salt and the compound **2** both soluble in the initial solutions. The use of polar protic solvents like methanol, ethanol, or water was instead prevented by the observation that molecule **2** did not form the complex in these solutions at the same 1.5 × 10^−4^ M concentration. We observed different behaviors between the free ligand (in dichloromethane and acetonitrile, obtaining comparable absorption profiles) and the chelate in dichloromethane and acetonitrile (Figure S1). In both cases, the stoichiometry of the metal–ligand complex was determined from the continuous variation method consisting of a photometric titration (Job’s method) [15]. In dichloromethane solution, the stoichiometry of ligand **2/**zinc(II) ion resulted 3:1 (Figure S2), whereas in acetonitrile it was 1:1. The same stoichiometry was obtained in acetonitrile for the Cu(II) and Mn (II) chelates (Figure 2).

The structure of Zn chelate **8** can find support based on the metal–ligand stoichiometry determined for the [ZnCl_2_(en)] complex (where en = 1,2-diaminoethane or ethylenediamine), in which a higher coordination involving more chloride ions was only obtained in the presence of an excess of chloride ions (NaCl) [16].

We have also investigated the chelation using NMR analysis. The first visual evidence of complexation was that the Zn-complex solutions assumed a more intense purple color in comparison with compound **2** at the same concentrations during the preparation of deuterated solutions. Furthermore, the metal coordination site in the structure of ligand **2** was determined from the chemical shift variations in the ^1^HNMR spectra of the preformed complex and the free molecule **2**, acquired in CDCl_3_ with a small amount of CD_3_OD added, and CD_3_OD solutions (Table 1). After complexation, a downfield shift is observed, with the most prominent value for the H-2 signal, which is the nearest proton of the *N* atom involved in the chelation. As expected, the change in the electronic environment caused by *N* chelation with the metal ions determines a deshielded shift in the proton on the carbon directly attached to the nitrogen. Its signal appears at a higher chemical shift, specifically at 0.20 ppm and 0.33 ppm in the CD_3_OD and CDCl_3_ + 2%CD_3_OD solutions, respectively (Table 1, Figure S3). Of note, the same signal shifts were evident in the ^1^HNMR spectrum when the preformed complex, created by treating two molar equivalents of ligand **2** with one molar equivalent of zinc chloride, was analyzed in CDCl_3_ at the same 4 mg/mL concentration.

Based on the correlations between the NMR shifts and the charge of the atoms, we used a computational approach to support this experimental observation. Among the computational tools for charge analysis, we selected the Voronoi deformation density (VDD) method, which is particularly efficient for calculating atomic charges in molecules and metal complexes [17]. The data showed an increased positive charge on the hydrogen atoms after metal chelation, with higher values for H-2 (+ 0.513 and + 0.536 in CDCl_3_ and CD_3_OD, respectively), in line with experimental NMR findings. Notably, the values calculated for the zinc atom (+ 0.3885 and + 0.3946) indicate a partially polarized covalent bond with chlorine atoms. If the less electronegative atoms become positively charged, the atoms with a higher electronegativity receive a negative charge. This behavior also happens for the N-1 and the oxygen in C-12. A positive difference in the charges in the metal complex supports the *N*/*O* chelation.

Additionally, we analyzed the effect of chelation in conditions more similar to the solution used in the antibacterial tests. The preformed Zn complex derived by complexing ligand **2** and chloride salt in a 1:1 molar ratio was acquired in D_2_O/DMSO-d_6_ (5:1) and compared with free ligand **2** in the same conditions. We observed comparable signal shifts as in the CDCl_3_ and CD_3_OD solutions (Figure S3).

**Figure 3:**
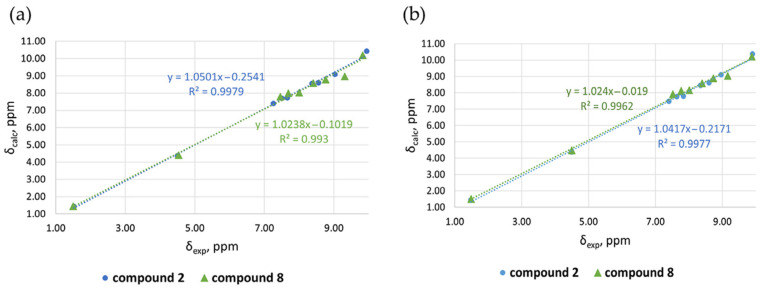
Correlation plots of calculated vs. experimental ^1^H NMR chemical shifts for free ligand **2** and Zn complex (**8**), in (**a**) chloroform and (**b**) methanol.

After carefully selecting the best combination of functional and basis set, we carried out DFT calculations at the M06-L/x2c-TZVPAll-s level of theory. A good linear fit was obtained between the experimental and calculated chemical shifts for the protons in the structure of Zn complex (**8**), validated by data obtained for free ligand **2** (Table 2, Figure 3).

Contrary to zinc, which is diamagnetic, and for which sharp signals were obtained for complex **8**, NMR analysis could not be performed for the copper and manganese complexes. In detail, paramagnetic copper(II) complexes can cause significant line broadening due to the unpaired electrons, which can affect the relaxation times of nearby protons, leading to broad, shifted signals that may be difficult to interpret. Similarly, manganese complexes with certain oxidation states, including Mn(II), are often paramagnetic, which broadens and obscures ^1^HNMR signals (Figure S4).

IR spectroscopic analysis has also confirmed the involvement of C12(=O) in metal chelation by comparing its value in compound **2** with those in complexes **8**–**10**. Density functional theory (DFT)-calculated spectra allowed us to assign the stretching vibrations of the carbonyl groups (Table 3, Figures S5–S8), which agree with the experimental ones. The absorbance frequencies of the carbonyl at the C-5 position mainly overlap with the contribution of the ester group. In contrast, the stretching vibrations corresponding to the carbonyl at the C-12 are in a distinct region, although a delocalized vibration of other moieties is present due to the fused structure of ligand **2**. Absorptions at lower wavenumber values are observed after chelation due to a decrease in the double bond feature, as supported by the distances C12-O and O–metal calculated for the minimized structures. These effects become more evident in the series of **8**–**10**, moving from zinc to manganese.

The metal complexes’ compositions were further studied by mass spectrometry, where the soft feature of electrospray ionization (ESI) allows the detection of metal–ligand systems. After direct infusion of a methanol solution of the preformed Zn complex, the spectrum acquired in positive ion mode showed a cluster signal around *m*/*z* 419 attributable to the [M-Cl]^+^ ion. The isotopic distribution was compared with the simulated data (Figure S9) and agrees with the 1:1 stoichiometry of the species established by UV–Visible measurements. The minor cluster signal around *m*/*z* 739 could be attributed to the complex with 2:1 ligand/Zn stoichiometry. Even the analysis of the complex produced by using a 2:1 ligand-to-metal ratio displayed the same spectrum, with the *m*/*z* 419 signal, corresponding to structure **8**, as the most intense and predominant one. Similarly to the data obtained for the Zn complex, the other analog chelates **9**–**10** also provided the cluster ion corresponding to the [M-Cl]^+^ species as the prevalent signal (Exper.). The calculated isotope mass distribution closely matches the experimental set of peaks (Figure S9).

No substantial changes were detected when analyzing the complexes dissolved in methanol or acetonitrile/water solutions, and no clusters were detected with molecules of water or methanol used as the solvent.

It has been reported that in most cases, the peaks observed in ESI-MS of metal complexes correspond with the species detected by other techniques, including UV-Vis measurements [18]. However, the ions produced can react in the gas phase before reaching the analyzer, creating artifacts. A series of processes inside the electrospray chamber may change the composition of the initial solution, affecting the number, the stoichiometry, and the relative abundance of the species. ESI-MS measurements are sensitive to the instrumental parameters employed, especially the cone voltage, which is used to remove solvent molecules attached to ions. In particular, gas-phase reactions are less likely to occur at low cone voltages, while a higher cone voltage can be used to fragment the ions [18]. By using the same concentration of the starting methanol solution of complexes **8**−**10**, we performed experiments by substantially changing the cone voltage applied. In detail, moving from 38.3 V to 51.8V, we always observed the major cluster corresponding to the 1:1 stoichiometry of the ligand/metal ion species, and an increased intensity of the minor cluster corresponding to a 2:1 ratio, attributable to a dissociation of the 1:1 complex, producing a ligand that can contribute to the 2:1 cluster.

### 2.3. Biological Evaluation

While *B. subtilis* is present in the human gut and is not particularly dangerous, *B. cereus*, *P. aeruginosa*, and *E. coli* are classified as pathogenic strains with the potential to develop antibiotic resistance, and hence are of interest in evaluating the antimicrobial activities of the tested compounds. As represented in Table 3, none of the compounds **1**–**7** showed activity toward the Gram-negative *P. aeruginosa* and *E. coli*. At the same time, the free ligand **2** was the most active against the Gram-positive *B. subtilis* and *B. cereus*. This activity was much higher and similar to vancomycin, respectively.

Complexes **8**–**10** provided the same degree of inhibition against *B. subtilis*, although with higher MIC values than the free ligand **2**, which was the most active in the series. Similar MIC values against *B. cereus* were observed in the cases of compound **2** and Mn complex **10**, and these were comparable to the activity of the control antibiotic vancomycin. Notably, among analogs **1**–**7**, the only compound active against the *Bacillus* strains besides **2** was Zn complex **8**, which showed the same *N*,*N*-syn structure. In contrast, the *N*,*N*-anti regioisomers **3** and **6** were inactive.

Further, none of compounds **1**–**10** exerted antibacterial activity against *P. aeruginosa*, or analogs **1**–**7** against *E. coli*. Among the complexes **8**–**10** that were tested for *E. coli* inhibition, the Cu chelate **9** was the most active, displaying an effect similar to penicillin G, which was used as the control antibiotic.

The qualitative structure–activity relationship of compounds **1**–**10** tested against *S. aureus* provided a behavior somewhat similar to that against the two *Bacillus* strains, indicative of a greater selectivity for Gram-positive over Gram-negative bacteria. Compound **2** was the most active among the tested compounds, with a MIC value 10 times lower than the other *N*,*N*-syn analog, **5**, and with slightly better activity than vancomycin, which was used as a control antibiotic (Table 4). These data once again confirmed the role of the *N*,*N*-syn configuration as an essential structural requirement in affecting the bioactivity. In the series of metal chelates **8**–**10**, the different metal ions were not so determinative in changing the biological effect, as evident by their comparable MIC values (1–2 µg/mL).

The evaluation of inhibitory activity against MRSA is of particular relevance due to the drug resistance of this strain. In this regard, the antibacterial activities of the tested compounds were evaluated against the standard MRSA strain ATCC 43300. In the preliminary experiment using the agar diffusion test, no effects on this strain were displayed by the inorganic chlorides used to produce the metal chelates (Table 5). This finding could exclude the toxic effect reported for specific metal species on their molecular targets. In fact, Zn(II) could cause toxicity by inhibiting the activity of proteins. Further, Cu(II) is a redox-active transition metal that can exert its toxicity by producing reactive oxygen species and antioxidant depletion, leading to impaired membrane function [5]. The largest inhibition zones caused by the Cu complex **9** indicated the most significant susceptibility among the tested compounds, where the lowest measurement was obtained for the free ligand **2**, resulting in a comparable value to Zn complex **8**. However, the values for all of them were still higher than the data that the antibiotics used as controls exhibited. Regarding the MIC values, we could obtain only preliminary results which prevented from establishing the exact lowest concentration that is required to inhibit bacterial growth in vitro.

A higher selectivity was obtained against the oxacillin-resistant *A. baumanii*, where only the Zn and Cu complexes allowed for measurements of the diameter of the inhibition zone. The MIC data obtained displayed the best value for Cu complex **9**. However, this value was still much higher than the antibiotics tested as positive controls (Table 5).

**Figure 4:**
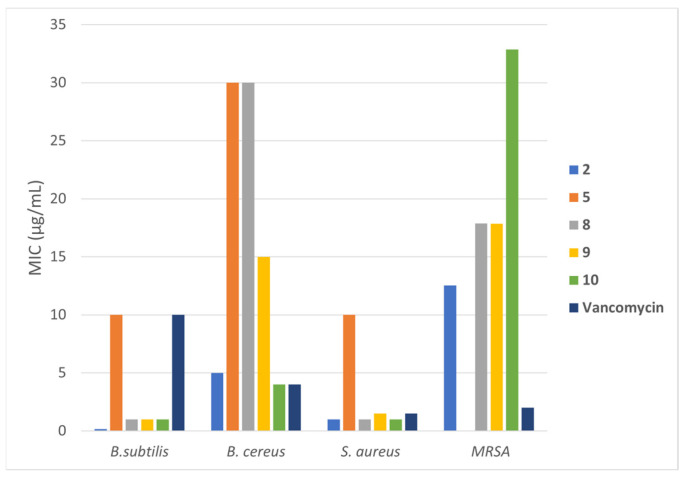
A summary of the most significant activities against the indicated strains by the molecules **2** and **5** and the metal complexes **8**–**10**.

Figure 4 displays a comprehensive comparative view of the strain inhibitions observed for the most active compounds compared to vancomycin.

### 2.4. Docking Calculations

To investigate the interactions of the most bioactive compounds with potential receptor sites of proteins related to bacterial infections, we performed in silico molecular docking using AutoDock 4.2.6 software.

**Figure 5:**
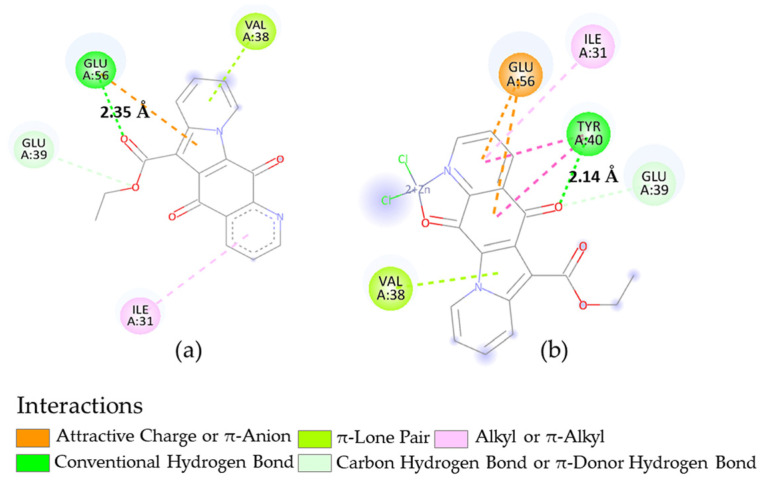
Two-dimensional representations for the interactions of compounds **2** (**a**) and complex **8** (**b**) in the receptor site of FtsZ (2VAM) with indication of interaction types and H-bond distance, as deduced by docking calculations.

We considered the FtsZ protein as a potential macromolecule of interest since it was reported in the literature as a validated key protein involved in the bacterial cell-division pathway in *B. subtilis*, resulting in a potential drug target [19]. The best binding energy value was obtained for compound **2**, within a non-significant range of variability compared to the corresponding zinc chelate **8** (Table 6). Docking result analysis showed that a hydrogen bond stabilized the protein complex in both cases, but different amino acid residues were involved, with more interactions for **8** (Figure 5). Therefore, it is evident that the presence of the zinc ion can induce a different disposition in this receptor site. These findings are in line with the experimental inhibitory activities.

We also analyzed a DNA gyrase, a topoisomerase enzyme involved in controlling topological transitions of DNA. It is essential for all bacteria, and its exploitation as a drug target has been reported to offer realistic therapeutic opportunities [20]. Moreover, gyrase is known as the target of quinolone antibiotics. Fluoroquinolones have been particularly successful in targeting gyrase, but increased antibacterial resistance has highlighted the need to investigate new mechanisms of action and develop new compounds [21]. Quinolones are characterized by a core structure constituted by a nitrogen-containing bicyclic planar scaffold similar to the indolizinoquinolinedione of the molecules here investigated. Therefore, the activity of *S. aureus* and MRSA related to gyrase led us to correlate the in vitro results with data from an in silico screening on this enzyme as a potential target.

**Figure 6:**
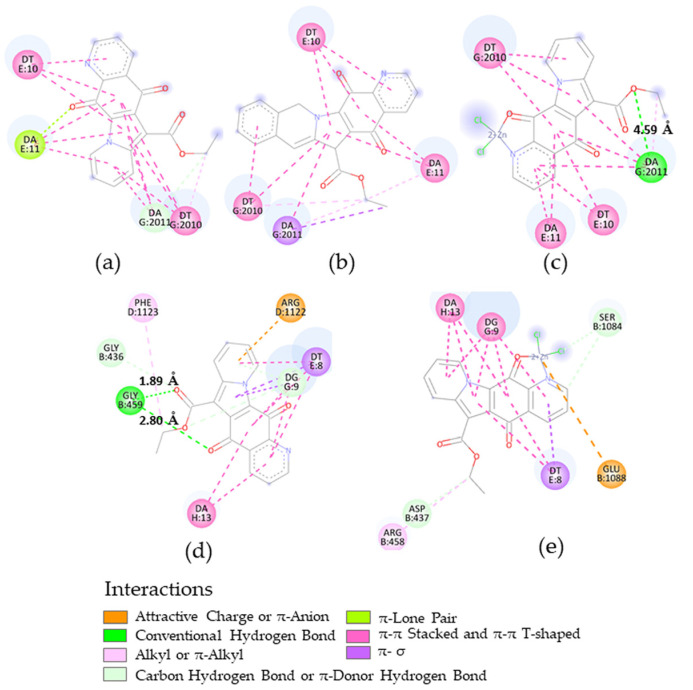
Two-dimensional representations for the interactions of the following: at the top, compounds **2** (**a**), **5** (**b**) and complex **8** (**c**) in the receptor site of (5IWI); at the bottom, compound **2** (**d**) and its zinc complex **8** (**e**) in the receptor site of DNA gyrase (2XCT), with indication of interaction types and H-bond distance, as deduced by docking calculations.

In detail, regarding the docking for *S. aureus* DNA gyrase (5IWI), the results for energy values are in the 2 kcal/mol reliability range (Table 6) and cannot discriminate between the ligands. Similar interactions involving DNA nucleotides are evident in both the *N*,*N*-syn regioisomers **2** and **5** and Zn chelate **8** as ligands. However, the most stabilizing hydrogen bond displayed by complex **8** is decisive in favoring its interaction (Figure 6a–c). Similar results (Table 6) were obtained considering the DNA gyrase from *S. aureus* N315, which was isolated as an MRSA (2XCT). Two favorable hydrogen bonds involving compound **2** were identified (Figure 6d,e), which is again consistent with the experimental data.

## 3. Materials and Methods

### 3.1. Chemistry

#### 3.1.1. General

All reagents and solvents were purchased from Sigma Aldrich and Merck (Avantor, Milan, Italy) and used without further purification. The compounds **2**–**7** were obtained and characterized as reported by the same authors [7,8,9,10]. A Shimadzu UV-1900 UV/vis spectrophotometer (Shimadzu Italia, Milan, Italy) was used to record UV spectra within the 620 to 300 nm range. The instrument used 0.2 nm increments and a fast speed. Quartz cuvettes with 1.0 cm path length and PTFE lid were used. The reference cell was always occupied with a cuvette of the same solvent without additives. The background spectra were recorded and checked with the blank solvent. For each measurement, the stock solutions were diluted within the cuvette to achieve an absorbance of 0.3–1.0 at 280 nm during recording. Infrared spectra were recorded by using a FT-IR Tensor 27 Bruker spectrometer (Bruker Optics GmbH, Karlsruhe, Germany) (Attenuated Transmitter Reflection, ATR configuration) at 1 cm^−1^ resolution in the absorption region 4000–600 cm^–1^. A thin solid layer was obtained by evaporation of a sample of methanol solution. Spectra were processed using the Opus software package (version 7.5.18). NMR spectra were recorded on a Bruker Avance 400 spectrometer using a 5 mm BBI probe ^1^H at 400 MHz and with values relative to TMS, in CDCl_3_ (δ 7.25 ppm) with 2%CD_3_OD added to increase the salt solubility, in CD_3_OD (δ 3.31 ppm), and in 5:1 D_2_O/DMSO-d_6_ (δ 2.49 ppm). Electrospray ionization (ESI)-MS mass spectra were recorded using a Bruker Esquire-LC spectrometer (Bruker Daltonic, Karlsruhe, Germany) by direct infusion of a methanol solution (source temperature 300 °C, drying gas nitrogen, 4 L·min^−1^, scan range *m*/*z* 100–1500). A software program (version 2.2) designed by the authors was used to obtain the simulated isotopic clusters reported in Figure S9.

#### 3.1.2. Synthesis and Structural Characterization of Metal Complexes

To a solution of compound **2** (10 mg, 0.031 mmol) in EtOH (1 mL), an equimolar amount of suitable metal chloride (ZnCl_2_, CuCl_2_∙2H_2_O, MnCl_2_∙4H_2_O) was added, and the mixture was refluxed for 5 h. The solvent was removed in vacuo, resulting in a purple powder, which was directly characterized and used in the biological evaluation. The complex was also formed without refluxing ethanol by simply evaporating the solvent used to dissolve the reagents. A similar procedure was adopted for producing the complex with ligand/Zn in a 2:1 ratio.

*(Ethyl 5,12-dihydro-5,12-dioxoindolizino[2,3-g]quinoline-6-carboxylate)zinc(II)chloride (**8**)*
  ^1^HNMR (400 MHz, CDCl_3_ + 2%CD_3_OD) δ 9.31(br. s, 1 H, H-2), 8.00 (dd, *J* = 8.0, 5.8 Hz, 1 H, H-3), 8.76 (d, *J* = 7.9 Hz, 1 H, H-4), 8.49 (d, *J* = 9.1 Hz, 1 H, H-7), 7.69 (br. t, *J* = 7.9 Hz, 1 H, H-8), 7.46 (br.t, *J* = 7.0 Hz, 1 H, H-9), 9.83 (d, *J* = 7.1 Hz, 1 H, H-10), 4.56 (q, *J* = 6.7 Hz, 2 H, CH_2_), 1.54 (t, *J* = 6.7 Hz, 3 H, CH_3_).
  FT-IR (cm^−1^): 3727 (w), 3427 (w), 1720 (m), 1684 (m), 1620 (s), 1570 (s), 1503 (m), 1478 (m), 1314 (s), 1227 (s), 1190 (m), 1127 (w), 1014 (w), 790 (w), 759 (w), 687(w). ESI(+)-MS: *m*/*z* 419 [Lig ^35^Cl^64^Zn]^+^, 739 [2Lig ^35^Cl^64^Zn]^+^, 343 [Lig+Na]^+^, 321 [Lig+H]^+^; ESI(+)MS/MS on 419: *m*/*z* 391, 347.
*(Ethyl 5,12-dihydro-5,12-dioxoindolizino[2,3-g]quinoline-6-carboxylate)copper(II)chloride (**9**)*
  FT-IR (cm^−1^): 3073 (w), 1686 (m), 1611 (s), 1576 (s), 1469 (m), 1306 (m), 1224 (vs), 1122 (m), 1084 (w), 1003 (w), 849 (w), 752 (s), 685 (w), 605 (w). ESI(+)-MS: *m*/*z* 418 [Lig ^35^Cl^63^Cu]^+^, 738 [2Lig ^35^Cl^63^Cu]^+^, 343 [Lig+Na]^+^; ESI(+)MS/MS on 418: *m*/*z* 390; on 738: *m*/*z* 418.
*(Ethyl 5,12-dihydro-5,12-dioxoindolizino[2,3-g]quinoline-6-carboxylate)manganese(II)chloride (**10**)*
  FT-IR (cm^−1^): 3398 (w), 3107 (w), 2971 (w), 1684 (s), 1617 (s), 1578 (s), 1474 (s), 1408 (w), 1306 (m), 1227 (vs), 1189 (s), 1127 (w), 1080 (w), 1031 (w), 761 (m), 691 (w); ESI(+)-MS: *m*/*z* 410 [Lig ^35^Cl^55^Mn]^+^, 730 [2Lig ^35^Cl^55^Mn]^+^, 343 [Lig+Na]^+^.

#### 3.1.3. Stoichiometry Determination of the Metal Complexes 8–10 from Spectrophotometric Titrations

The stoichiometry of the complex was determined using the Job’s plot method. For a complex represented as *M*_n_*L*_m_ (where *M* is the metal ion and *L* the ligand), the equilibrium formation can be described by the following equation:

(1) $$nM+mL\;⇌M_{n}L_{m}$$

with the stability constant given by the following:

(2) $$\beta_{nm}=\frac{{[M}_{n}L_{m}]}{\left[M^{n}\right][L^{m}]}$$

The absorbance (Abs) of a series of solutions containing the metal ion and the ligand is measured, keeping the sum of their total concentrations constant:

(3) $$C_{M}+C_{L}=C=Costant$$

The position of the absorbance maximum (Abs_max_) is then related to the stoichiometric ratio *n*/*m.* Using molar fraction *X*, defined as follows:

(4) $$X=\frac{C_{M}}{C_{M}+C_{L}}$$

the stoichiometric ratio *n*/*m* could be calculated from *X_max_* through the following relationship:

(5) $$\frac{n}{m}=\frac{X_{max}}{1-X_{max}}$$

The value of *X_max_* (corresponding to Abs_max_) is determined from the maximum absorbance recorded at a suitable wavelength, selected to ensure that absorbance changes are as pronounced as possible.

### 3.2. Biological Evaluation

#### Determination of Antibacterial Activity

Antibacterial activity was assessed against Gram-positive *B. subtilis* (EXB-V68), *B. cereus* (EXB-L-432), and *S. aureus* (EXB-V54), and Gram-negative *E. coli* (EXB-V1) and *P. aeruginosa* (EXB-V28), by using the standard agar-diffusion test. The bacterial strains were obtained from the Infrastructural Centre Mycosmo (Chair for Molecular Genetics and Biology of Microorganisms, Department of Biology, Biotechnical Faculty, University of Ljubljana). After growing the bacteria overnight in Luria–Bertani (LB) medium and determining their concentration by a turbidimetric test, an appropriate volume of bacterial culture was added to LB nutrient agar previously cooled to 42 °C to obtain approximately 5 × 10^5^ CFU/mL. Twenty milliliters of the inoculated medium were poured into Petri dishes and incubated for 24 h at 4 °C. Then, circles of agar (F = 1 cm) were cut from the cooled media, and 100 μL of solutions of different compounds or antibiotics (positive controls) were pipetted into the holes. The plates were incubated for 24 h at 37 °C; afterwards, the diameters of inhibition zones were measured. Penicillin, amoxicillin, and vancomycin, used as control antibiotics, were dissolved in deionized water, and synthetic compounds were dissolved in DMSO at 10 mM concentration (stock solutions). Serial dilutions of DMSO-dissolved stock solutions were made in deionized water and tested as negative controls. Every measurement was repeated in three parallel samples. DMSO had antibacterial effects only when tested undiluted, and only on *P. aeruginosa.* The MIC values (in μg/mL) reported in Table 4 correspond to the lowest concentration of antibiotic causing visible inhibition of bacterial growth.

Antimicrobial susceptibility assay against MRSA (ATCC 43300) and *A. baumanii* (oxacillin-resistant, OXAR), a hospital-origin strain belonging to the Applied Microbiology Laboratory at the University of Bejaia, Algeria, was performed using the well-diffusion method. Wells of 6 mm in diameter were formed on Mueller–Hinton agar plates, pre-inoculated with 10^7^ CFU/mL of MRSA and *A. baumanii*. All tested compounds were prepared at a concentration of 10 mM, and 20 μL of each was introduced into the wells. DMSO was a negative control, while vancomycin, tetracycline, gentamycin, and cefotaxime were positive controls. The plates were placed at 4 °C for 2 h to allow for the diffusion of the substances before being incubated at 37 °C for 24 h. The clear zones of inhibition observed around the wells suggested antagonistic activity, and diameters of inhibition zones were subsequently measured. The experiment was performed in duplicate. The minimum inhibitory concentration (MIC) of the active compounds was determined using the serial dilution method in 96-well microtiter plates, as defined by the Clinical and Laboratory Standards Institute (CLSI 2020) guidelines [22].

The compounds were tested against pathogenic microorganisms at final concentrations of 10 mM, ranging from 2250 μg/mL to 17.50 μg/mL for each metal chelate **8**–**10** and from 1601.5 μg/mL to 12.53 μg/mL for free ligand **2**, with DMSO, distilled water, and Mueller Hinton broth used as negative controls. After incubation, the MIC was determined as the lowest concentration visibly inhibiting the growth of each tested microorganism [22].

### 3.3. Computational Analysis

#### 3.3.1. DFT Calculation

Calculations were carried out on a PC running at 3.4 GHz on an AMD Ryzen 9 5950X 16-core (32 threads) processor with 32 GB RAM and 1 TB hard disk with Windows 10 Home 64-bit as an operating system. Compounds **1**–**7** were built using PC Model version 10 (Serena Software, Bloomington, IN, USA, IN 47402e3076) and pre-minimized using the force field MMX. The geometry was subsequently optimized by quantum chemical calculations using the Gaussian 16W revision A.03 package program set [23]. Restricted mode was used, except for the copper and manganese complexes, for which unrestricted mode was applied, and performed in vacuo for geometry optimization. The basis set of choice was 6-311+G(d,p). The gradient-corrected DFT with the three-parameter hybrid functional (B3) [24] for the exchange part and the Lee–Yang–Parr correlation function [25] with the D3 version of Grimme’s dispersion added, with Becke–Johnson damping empirical dispersion (GD3BJ) [26] utilized and the optimized structural parameters were employed in the vibrational energy calculations at the same DFT levels to characterize all stationary points as minima, also used in IR spectra simulation. The calculated IR frequencies were scaled by 0.964. No imaginary wavenumber modes were obtained for the optimized structure, proving that a local minimum on the potential energy surface was found. All the minimized molecules were saved in a pdb extension. Population analysis and atomic charges calculation were performed by Multiwfn software (Version: 3.8(dev), update date: 13 April 2025) [27,28], choosing the Voronoi deformation density (VDD) atom population. For NMR simulation, the structures **2** and **8** were minimized as described above, in chloroform and in methanol, via a Conductor-like Polarized Continuum Model (C-PCM), obtaining no vibrational imaginary wavenumber modes as an indication of a reached minimum in the potential energy surface. The NMR methods applied in our study were the gauge-including atomic orbital (GIAO) [29] using the meta-generalized gradient approximation (M-GGA) M06L functional [30,31] in combination with the relativistic x2c-TZVPAll-s basis set [32]. The isotropic shift constants (σ) were obtained for each nucleus, and these were converted to a chemical shift (δ) value according to the equation δi = σTMS − σi. The reference substance was tetramethylsilane (TMS), calculated at the same level of theory.

#### 3.3.2. Molecular Docking

The AutoDock Tools (ADT) package version 1.5.6rc3 [33] was applied to generate the docking input files used for the calculations. The crystallographic structures of bacterial DNA gyrase and FtsZ proteins were obtained from the Protein Data Bank (PDB; https://www.rcsb.org/) and were: *S. aureus* DNA gyrase (PDB-ID: 5IWI) in complex with inhibitor (*1R*)-1-[(4-{[(6,7-dihydro[1,4]dioxino[2,3-c]pyridazin-3-yl)methyl]amino}piperidin-1-yl)methyl]-9-fluoro-1,2-dihydro-4H-pyrrolo[3,2,1-ij]quinolin-4-one (GSK945237) at resolution = 1.98Å [34], *S. aureus* isolated as an MRSA DNA gyrase (PDB-ID: 2XCT) in complex with inhibitor ciprofloxacin at resolution = 3.35 Å [35], *E. coli* DNA gyrase B (PDB-ID: 6YD9) in complex with N-[6-(3-azanylpropanoylamino)-1,3-benzothiazol-2-yl]-3,4-bis(chloranyl)-5-methyl-1H-pyrrole-2-carboxamide at resolution = 1.60 Å [36] and *B. subtilis* FtsZ protein (PDB-ID: 2VAM) at resolution = 2.50Å [37]. The structures were modified as follows: the ligand and all the crystallized water molecules were removed, with the file saved as a pdb extension. All hydrogen atoms were added using AutoDock Tools (ADT), and the Gasteiger–Marsili charges were calculated, with the resulting file saved in a pdbqt extension. Rotatable bonds were defined for each minimized ligand molecule. For the docking calculation, a grid box of 40 × 40 × 40 number of points in the x, y, and z directions was created for all the DNA-gyrase enzymes with spacing of 0.375 Å and centered at x = 5.496, y = 44.071, z = 40.433 for PDB-ID: 5IWI; x = 2.968, y = 43.484, z = 68.575 for PDB-ID: 2XCT; and x = 10,775, y = -5.034, z = 6.745 for PDB-ID:6YD9, whereas for *B. subtilis*, FtsZ protein (PDB-ID: 2VAM) adopted a blind-docking procedure with a grid box of 126 × 126 × 126 number of points with spacing of 0.375 Å, centered on the macromolecule at x = 28.981, y = -8.989, and z = −1.993. The following parameters were used in docking calculations: number of independent Genetic Algorithm (GA) Runs = 10; population size = 150; maximum number of evaluation = 2,500,000; maximum number of generations 27,000; maximum number of top individuals that automatically survive = 1; rate of gene mutation = 0.01; rate of crossover = 0.8; genetic algorithm crossover mode = twopt; mean of Cauchy distribution for gene mutation = 0.0; variance of Cauchy distribution for gene mutation = 1.0; and number of generations to pick the worst individual = 10. To validate the calculation, the original ligand was re-docked, and visual inspection of the data showed very tight overlap. The results are expressed as the energy associated with each ligand–enzyme complex in terms of the Gibbs free energy values. The visual ligand–enzyme interactions were displayed using Biovia Discovery Studio Visualizer (v21.1.0.20298) [38].

## 4. Conclusions

In this study, the indolizinoquinolinedione analogs **1**–**7**, whose synthesis was described in our previous reports, were evaluated for their activity against a series of bacteria. Minimal inhibitory concentration values indicated that *N*,*N*-syn **2** and **5** were more active than the corresponding *N*,*N*-anti regiosiomers **3** and **6**. Moreover, analog **2** was the most active against *B. subtilis*, *B. cereus*, *S. aureus*, and MRSA, and was therefore used as the ligand for producing new metal complexes.

The structures of the complexes were mainly analyzed in solution to reproduce the conditions of their biological application in more detail. The Zn-chelate stoichiometry was established by Job’s plot built through UV-vis analysis in solvents of different polarities, indicating a ligand/metal ratio of 1:1 in acetonitrile solution. ^1^HNMR data obtained for the complex and free ligand **2** in different solvents were correlated to the DFT-calculated atomic charge distribution affected by metal chelation. For complexes **8**–**10**, the fact that C=O was involved in the coordination was confirmed by FT-IR spectroscopy, with the assignments supported by DFT-calculated spectra. Additionally, ESI-MS analysis in the positive ion mode of the preformed complexes at both 1:1 and 2:1 ligand/metal chloride ratios provided significant isotopic clusters supporting a 1:1 chelation.

From the in vitro test, similar activities from **8**–**10** were observed against *B. subtilis*, although they were lower than that of the free ligand **2**. Comparable effects of **2** and **8**–**10** with vancomycin as a control antibiotic were demonstrated against *S. aureus.*

Moreover, FTsZ protein, as a potential target of *B. subtilis*, and DNA gyrase, as a potential target of *S. aureus* and MRSA, were studied by docking calculations, showing a good correlation with the in vitro results.

## Figures and Tables

**Table 1:** Chemical shifts in ligand **2** in free form and in the preformed Zn complex by ^1^HNMR spectra acquired in CDCl_3_ and in CD_3_OD at a concentration of 4 mg/mL, and values of atomic charges calculated by Voronoi deformation density (VDD) method.

Exptl δ(ppm)				Calcd. Charges		
In chloroform						
Position ^1^	**2** ^2^	Zn complex ^2^	Δδ	**2**	Zn complex	Δq
H-2	8.98	9.31	+0.33	0.0684	0.1197	+0.0513
H-3	7.66	8.00	+0.34	0.0765	0.0932	+0.0167
H-4	8.54	8.76	+0.22	0.0801	0.0960	+0.0159
H-7	8.34	8.49	+0.15	0.0793	0.0890	+0.0097
H-8	7.49	7.69	+0.20	0.0763	0.0859	+0.0096
H-9	7.26	7.46	+0.20	0.0802	0.0894	+0.0092
H-10	9.93	9.83	-0.10	0.0844	0.0999	+0.0155
Zn	-	-	-	-	0.3885	-
N-1	-	-	-	−0.1789	−0.0763	+0.1026
O in C-12	-	-	-	−0.3077	−0.2084	+0.0993
In methanol						
Position ^1^	**2** ^2^	Zn complex ^2^	Δδ	**2**	Zn complex	Δq
H-2	8.96	9.16	+0.20	0.0702	0.1238	+0.0536
H-3	7.84	8.01	+0.17	0.0809	0.0978	+0.0169
H-4	8.60	8.73	+0.13	0.0814	0.0986	+0.017
H-7	8.35	8.40	+0.05	0.0798	0.0899	+0.0101
H-8	7.64	7.77	+0.13	0.0804	0.0892	+0.0088
H-9	7.40	7.52	+0.12	0.0832	0.0926	+0.0094
H-10	9.90	9.88	+0.02	0.0846	0.0999	+0.0153
Zn	-	-	-		0.3946	-
N-1	-	-	-	−0.1878	−0.0738	+0.1140
O in C-12	-	-	-	−0.3149	−0.2177	+0.0972

^1^ Numbering as reported in Figure 1. ^2^ In CDCl_3_ added of 2%CD_3_OD.

**Table 2:** Experimental and calculated proton chemical shifts (in ppm) in compound **2** and Zn complex (**8**) in chloroform and methanol.

	Chloroform			Methanol		
**Position** ^1^	**Exptl.**	**Calcd.**	**|Δ|**	**Exptl.**	**Calcd.**	**|Δ|**
	Compound **2**					
H-10	9.94	10.42	0.48	9.90	10.37	0.47
H-2	9.03	9.09	0.06	8.96	9.10	0.14
H-4	8.56	8.60	0.04	8.60	8.61	0.01
H-7	8.37	8.55	0.18	8.35	8.46	0.11
H-3	7.66	7.72	0.06	7.84	7.79	0.05
H-8	7.50	7.69	0.19	7.64	7.77	0.13
H-9	7.26	7.40	0.14	7.40	7.48	0.08
CH_2_	4.50	4.37	0.13	4.49	4.39	0.10
CH_3_	1.52	1.43	0.09	1.48	1.43	0.05
MAE ^2^			0.15			0.13
	Compound **8**					
H-10	9.83	10.19	0.36	9.88	10.21	0.33
H-2	9.31	8.96	0.35	9.16	9.03	0.13
H-4	8.76	8.78	0.02	8.73	8.88	0.15
H-7	8.41	8.58	0.17	8.40	8.57	0.17
H-3	8.00	8.02	0.02	8.01	8.15	0.14
H-8	7.69	7.98	0.29	7.77	8.12	0.35
H-9	7.46	7.78	0.32	7.52	7.91	0.39
CH_2_	4.53	4.4	0.13	4.5	4.48	0.02
CH_3_	1.50	1.44	0.06	1.49	1.51	0.02
MAE ^2^			0.19			0.19

^1^ Assignments according to the numbering reported in Figure 1. ^2^ Minimal absolute error.

**Table 3:** Wavenumber values (cm^−1^) of the C=O groups in molecule **2** and metal complexes **8**–**10** by experimental IR spectra and DFT-calculated spectra at the B3LYP-GD3BJ/6-311+G(d,p) level of theory.

Compound	C-12(=O)		C-5(=O) + COOEt	d(Å) in the Minimized Structure
2	Calcd.	1642	1678, 1671	C12-O = 1.22
	Exptl.	1641	1680, 1674	
8	Calcd.	1605	1687, 1679	C12-O = 1.24 O-Zn = 2.19, N-Zn = 2.18
	Exptl. Δν (2-Zn complex)	1620	1684, 1720	
		−0.21		
9	Calcd.	1602	1687, 1679	C12-O = 1.24 O-Cu = 2.17, N-Cu = 2.10
	Exptl. Δν (2-Cu complex)	1611	1692, 1686	
		−30		
10	Calcd.	1484	1608(keto), 1669 (ester)	C12-O = 1.32 O-Mn = 1.90, N-Mn = 1.94
	Exptl. Δν (**2**-Mn complex)	1475	1620,1683	
		−166		

**Table 4:** Antibacterial activity (MIC in µg/mL) of the analogs **1**–**7** and the metal complexes **8**–**10** with **2** as ligand on Gram-positive (*Bacillus subtilis*, *Bacillus cereus*, *Staphylococcus aureus*) and Gram-negative (*Escherichia coli*, *Pseudomonas aeruginosa*) bacterial strains, evaluated using the standard agar diffusion assay. The minimum inhibitory concentration (MIC) values correspond to the lowest concentration of the compound causing visible inhibition of bacterial growth.

	MIC (µg/mL)				
Compound	*B. subtilis*	*B. cereus*	*P. aeruginosa*	*E. coli*	*S. aureus*
**1**	>1000	>1000	1000	>1000	>1000
**2**	0.15 ± 0	5.0 ± 0.5	>1000	>1000	1.0 ± 0.3
**3**	>1000	>1000	1000	>1000	>1000
**4**	>1000	>1000	>1000	>1000	>1000
**5**	10 ± 1	30 ± 2.5	>1000	>1000	10.0 ± 0.8
**6**	>1000	>1000	>1000	>1000	100
**7**	>1000	>1000	1000	>1000	>1000
**8**	1.0 ± 0.2	30 ± 0	>1000	300	1.0 ± 0.2
**9**	1 ± 0	15 ± 1	>1000	150	1.5 ± 0.2
**10**	1.0 ± 0.4	4 ± 0.3	1000	1000	1 ± 0
Penicillin G	>1000	>1000	>1000	150	>1000
Vancomycin	10.0 ± 0.8	4 ± 0.5	>1000	>1000	1.5 ± 0.2
Amoxicillin	>1000	>1000	>1000	15 ± 1	>1000

**Table 5:** Antibacterial activity of compound 2, its metal chelates **8**–**10**, and the inorganic chlorides used to produce the metal chelates tested on MRSA (ATCC 43400) and *Acinetobacter baumanii* (oxacillin-resistant, OXAR). Diameters of inhibition zone (mm) were evaluated by the agar diffusion method, and minimum inhibitory concentration (MIC) values (µg/mL) were evaluated for the compounds that resulted in activity.

	MRSA (ATCC 43300)		*A. baumanii* (OXAR)	
	Diameter of Inhibition Zones (mm)	MIC (µg/mL)	Diameter of Inhibition Zones (mm)	MIC (µg/mL)
ZnCl_2_	0	-	0	-
CuCl_2_∙2H_2_O	0	-	0	-
MnCl_2_∙4H_2_O	0	-	0	-
**2**	21.7 ± 1.2	<12.53	0	-
**8**	23 ± 0	<17.89	9 ± 0	286.34
**9**	31 ± 0	<17.85	18.5 ± 0.7	35.70
**10**	26.5 ± 0.7	<32.86	0	-
Tetracycline (30 µg/mL)	16 ± 0	<2	14 ± 0	8
Gentamycin (10 µg/mL)	13 ± 0	<2	13 ± 0	4
Vancomycin (30 µg/mL)	14 ± 0	<2	-	-
Cefotaxime (30 µg/mL)	-	-	16.5 ± 0.7	16

**Table 6:** Data from docking calculations of compounds **2**, **5** and zinc complex **8** with the targets indicated, related to the corresponding microorganism.

Microorganism	Target	PDB-ID	Compound	Calcd. Binding ΔG (kcal/mol)
*B. subtilis*	FtsZ	2VAM	**2**	−7.35
			**8**	−6.73
*S. aureus*	DNA gyrase	5IWI	**2**	−7.24
			**5**	−7.71
			**8**	−6.60
*S. aureus* N315 isolated as an MRSA	DNA gyrase	2XCT	**2**	−8.79
			**8**	−8.58

## Data Availability

The original contributions presented in this study are included in the article/Supplementary Materials. Further inquiries can be directed to the corresponding authors.

## Supplementary Materials

The following supporting information can be downloaded at https://www.mdpi.com/article/10.3390/molecules31020348/s1: Figure S1: UV-visible spectra of free ligand **2** and upon addition of ZnCl_2_; Figure S2: Job’s plot of ligand **2**/Zn complex in dichloromethane; Figure S3: ^1^H-NMR spectra of **2** and its preformed zinc complex in the indicated solvents; Figure S4: ^1^H-NMR spectra of metal complexes; Figures S5–S8: Experimental FT-IR and DFT-calculated spectra of **2** and its metal complexes **8**–**10**; Figure S9: ESI(+) MS analysis of the complexes **8**–**10**: experimental and simulated isotopic clusters of the [M-Cl]^+^ ions.
