# Supplementary material for: Indolizinoquinolinedione Metal Complexes: Structural Characterization, In Vitro Antibacterial, and In Silico Studies
Source: Molecules. 2026 Jan 19;31(2):348. doi: 10.3390/molecules31020348 (PMC12843794; doi:10.3390/molecules31020348)
Supplement: Supplementary file 1 [file molecules-31-00348-s001.zip › molecules-3917450-Supplementary.pdf]

Supplementary Materials

Indolizinoquinolinedione Metal Complexes: Structural Characterization, *In Vitro* Antibacterial and *In Silico* Studies

Jacopo Vigna[1], Michael Marchesi[1], Ibtissem Djinni[2], Miša Mojca Cajnko [3,4], Kristina Sepčić [3], Andrea Defant [1,*] and Ines Mancini [1,*]

Table of Contents

Figure S1. UV-visible absorption spectra of free ligand **2** (overlapped profile for solution in dichloromethane and acetonitrile, yellow) and the change in its spectrum upon addition of $ZnCl_2$ salt, acquired in dichloromethane (light blue) and in acetonitrile (green).

Figure S2. Changes in absorbance during complex formation in dichloromethane, starting from the solutions of $ZnCl_2$ and the ligand, both at 1.5×10^{-4} M, prepared in dichloromethane and then mixed in different molar ratios (top); Job's plot: absorbance values as a function of the molar fraction *X* of the ligand **2** during the formation of the complex by adding $ZnCl_2$ in dichloromethane (λ_{abs} = 554 nm), (bottom).

Figure S3. ^{1}H-NMR spectra (400MHz, range 7-10 ppm) of the molecule **2** (green) and its preformed zinc complex (blu), in the indicated solvents at a 4 mg/mL concentration.

Figure S4. ^{1}HNMR spectra acquired in $CDCl_3$ for the same amount of complexes and the same number of acquired transients, obtaining the following width values at half maximum of chloroform signal: 12 Hz for Cu-complex, 63 Hz for Mn-complex, 1.9 Hz for Zn-complex, and 0.9 Hz for chloroform.

Figure S5. FT-IR spectrum and DFT-calculated spectrum at the B3LYP-GD3BJ/ 6-311+G(d,p) level of theory, with the energy-minimized structure of molecule **2**.

Figure S6. FT-IR spectrum and DFT-calculated spectrum at the B3LYP-GD3BJ/ 6-311+G(d,p) level of theory, with the energy-minimized structure of zinc complex **8**.

Figure S7. FT-IR spectrum and DFT-calculated spectrum at the B3LYP-GD3BJ/ 6-311+G(d,p) level of theory, with the energy-minimized structure of copper complex **9.**

Figure S8. Experimental FT-IR spectrum and DFT-calculated spectrum at the B3LYP-GD3BJ/ 6-311+G(d,p) level of theory, with the energy-minimized structure of manganese complex **10**.

Figure S9. ESI(+) MS analysis of the complexes **8–10**: experimental (black) and simulated (red) isotopic clusters of the $[M-Cl]^+$ ions, where M= complexes of the indicated metal ions.

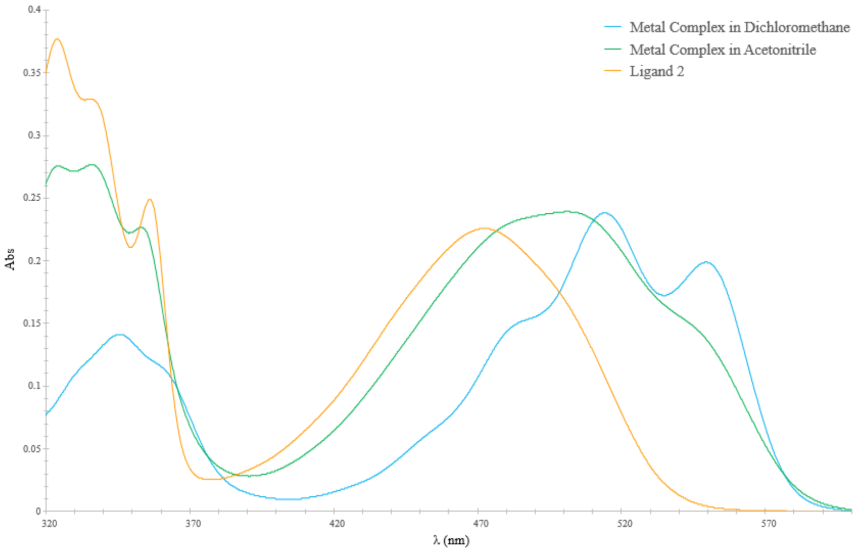

Figure S1. UV-visible absorption spectra of free ligand **2** (overlapped profile for solution in dichloromethane and acetonitrile, yellow) and the change in its spectrum upon addition of $ZnCl_2$ salt, acquired in dichloromethane (light blue) and in acetonitrile (green).

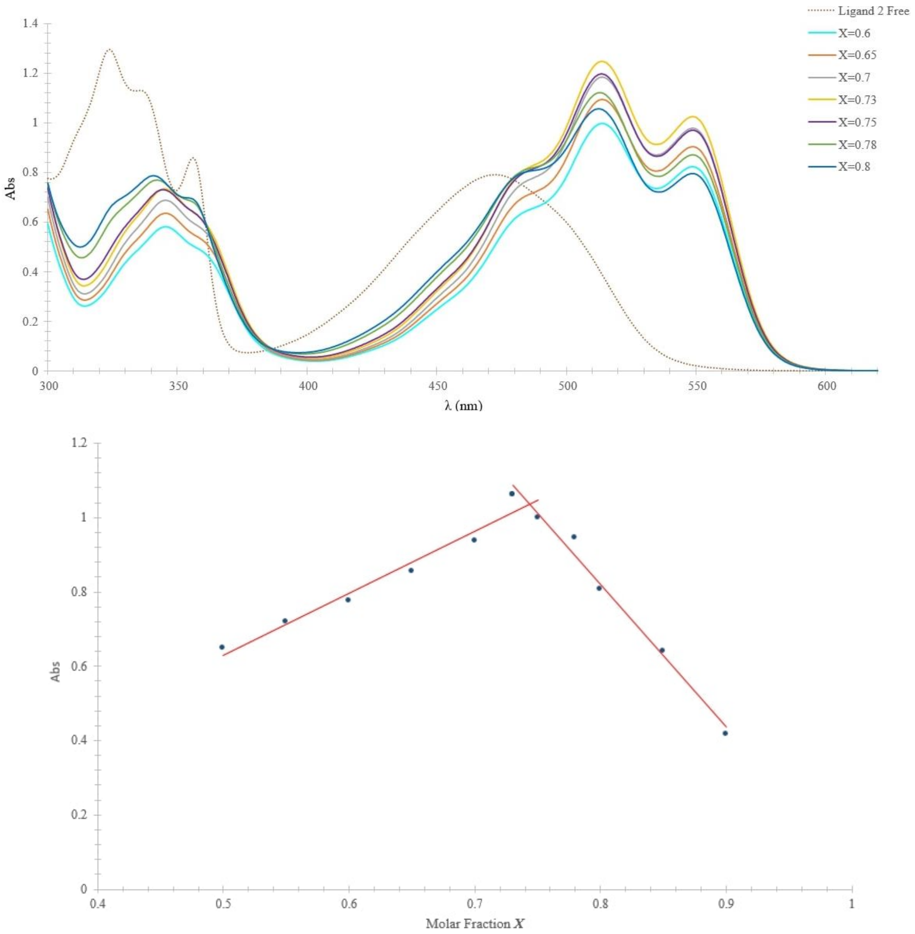

Figure S2. Changes in absorbance during complex formation in dichloromethane, starting from the solutions of $ZnCl_2$ and the ligand, both at 1.5×10^{-4} M, prepared in dichloromethane and then mixed in different molar ratios (top); Job's plot: absorbance values as a function of the molar fraction X of the ligand **2** during the formation of the complex by adding $ZnCl_2$ in dichloromethane (λ_{abs} = 554 nm), (bottom).

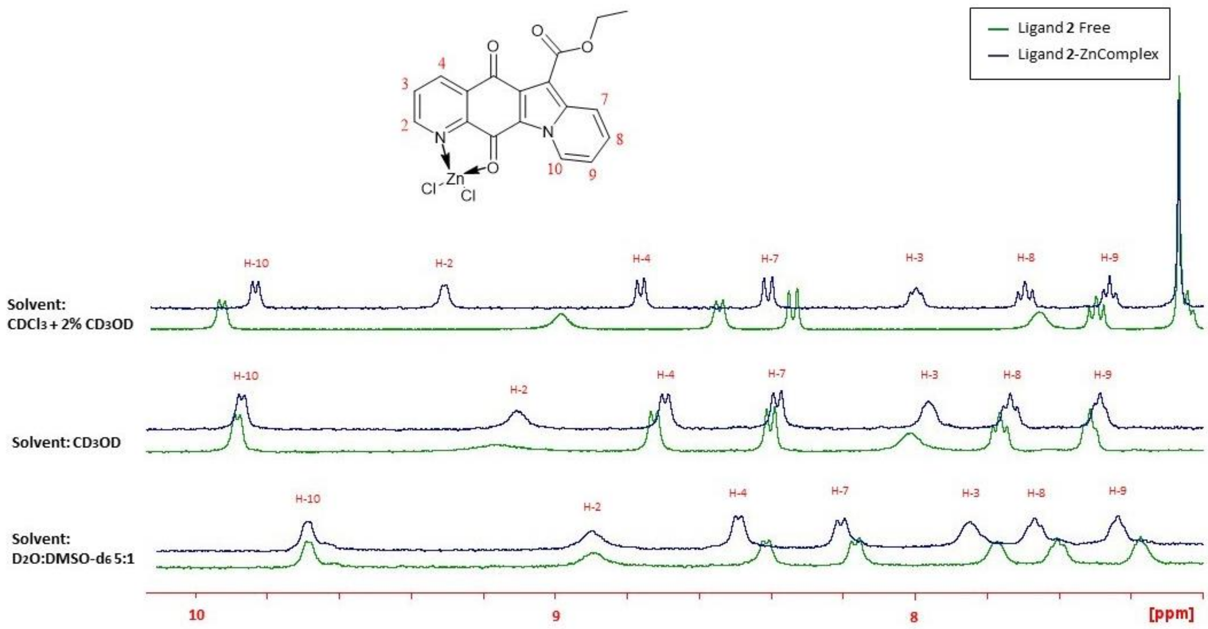

Figure S3. ^{1}H-NMR spectra (400MHz, range 7-10 ppm) of the molecule **2** (green) and its preformed zinc complex (blu), in the indicated solvents at a 4 mg/mL concentration.

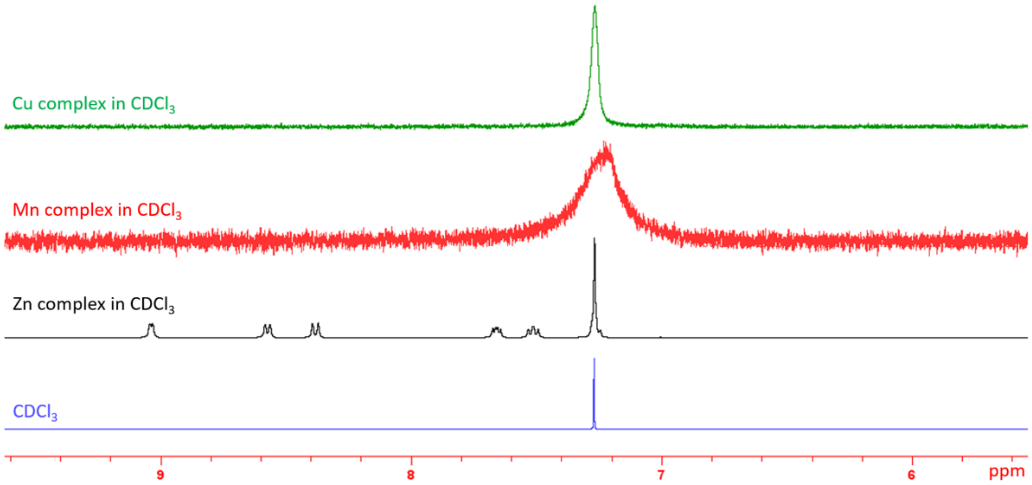

Figure S4. ^{1}HNMR spectra acquired in $CDCl_3$ for the same amount of complexes and the same number of acquired transients, obtaining the following width values at half maximum of chloroform signal: 12 Hz for Cu-complex, 63 Hz for Mn-complex, 1.9 Hz for Zn-complex, and 0.9 Hz for chloroform.

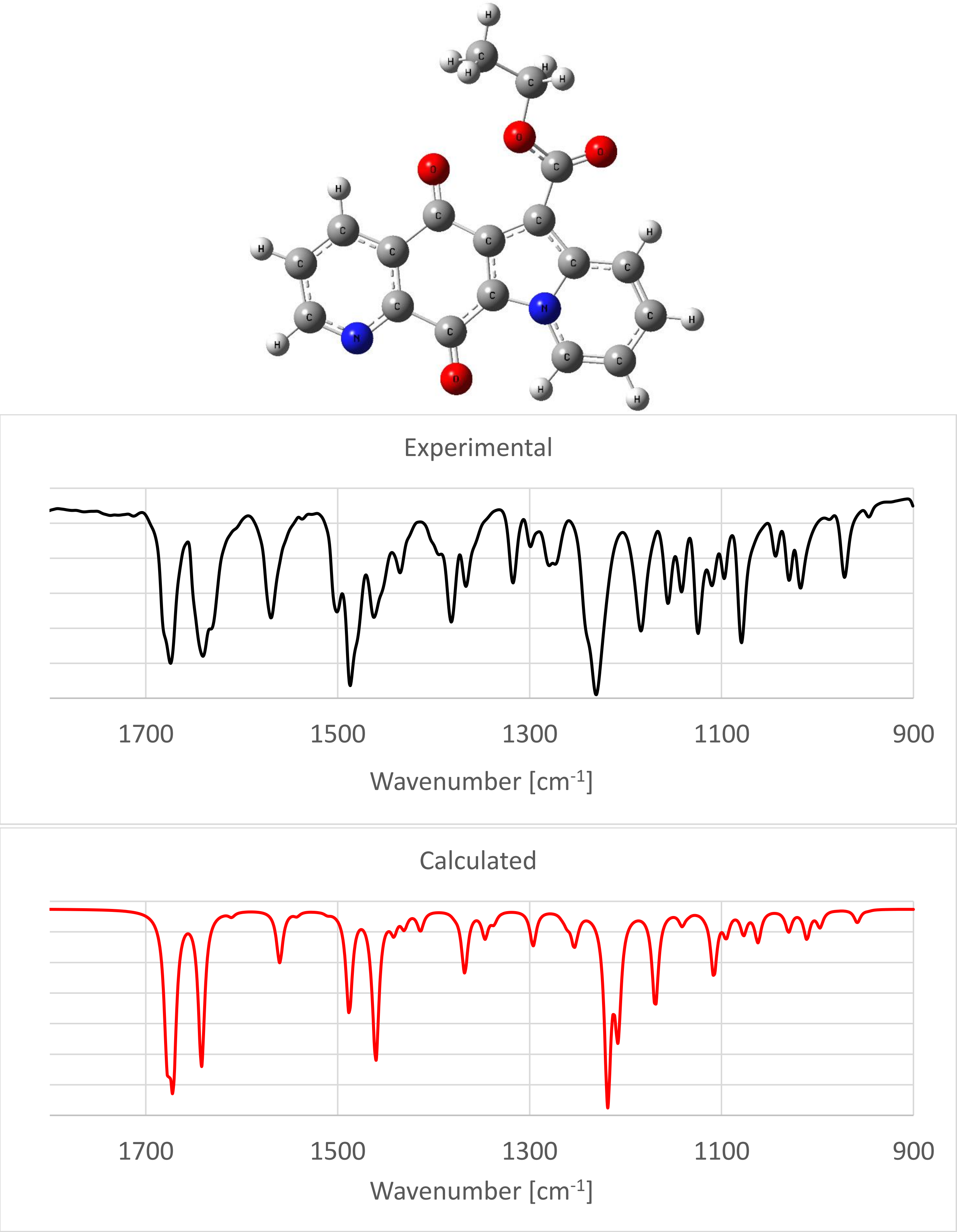

Figure S5. FT-IR spectrum and DFT-calculated spectrum at the B3LYP-GD3BJ/ 6-311+G(d,p) level of theory, with the energy-minimized structure of molecule **2**.

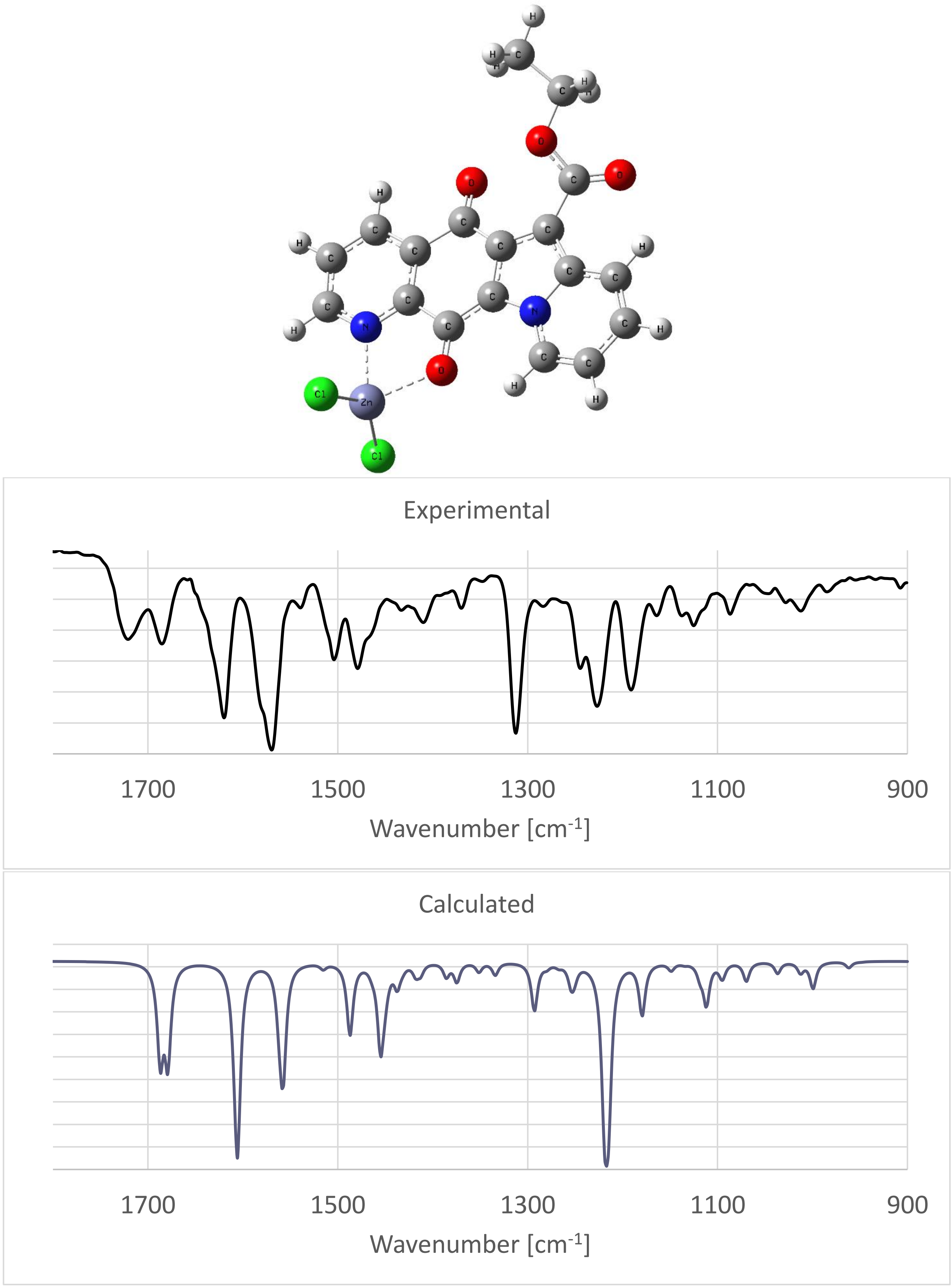

Figure S6. FT-IR spectrum and DFT-calculated spectrum at the B3LYP-GD3BJ/ 6-311+G(d,p) level of theory, with the energy-minimized structure of zinc complex **8**.

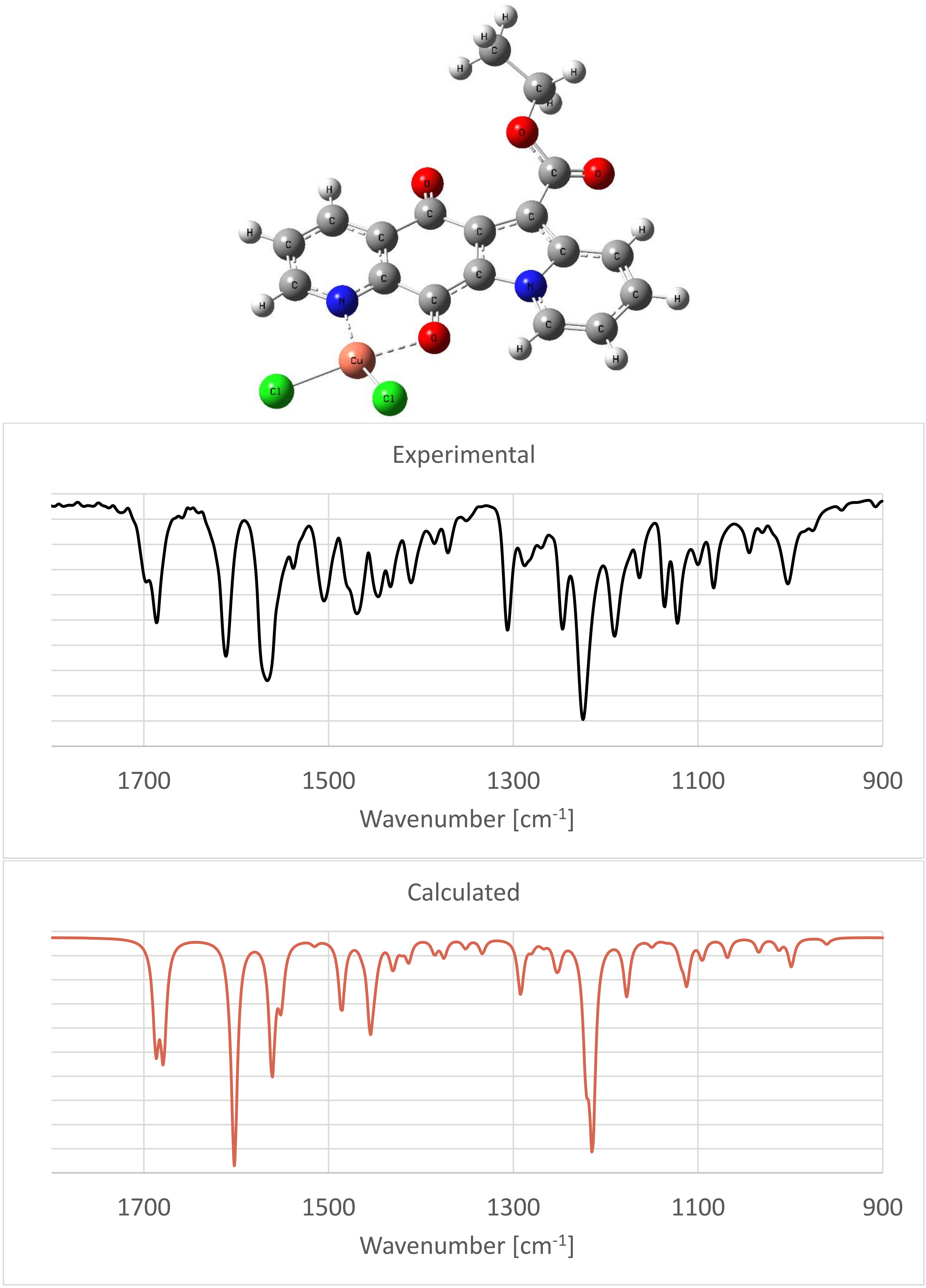

Figure S7. Experimental FT-IR spectrum and DFT-calculated spectrum at the B3LYP-GD3BJ/ 6-311+G(d,p) level of theory, with the energy-minimized structure of copper complex **9**.

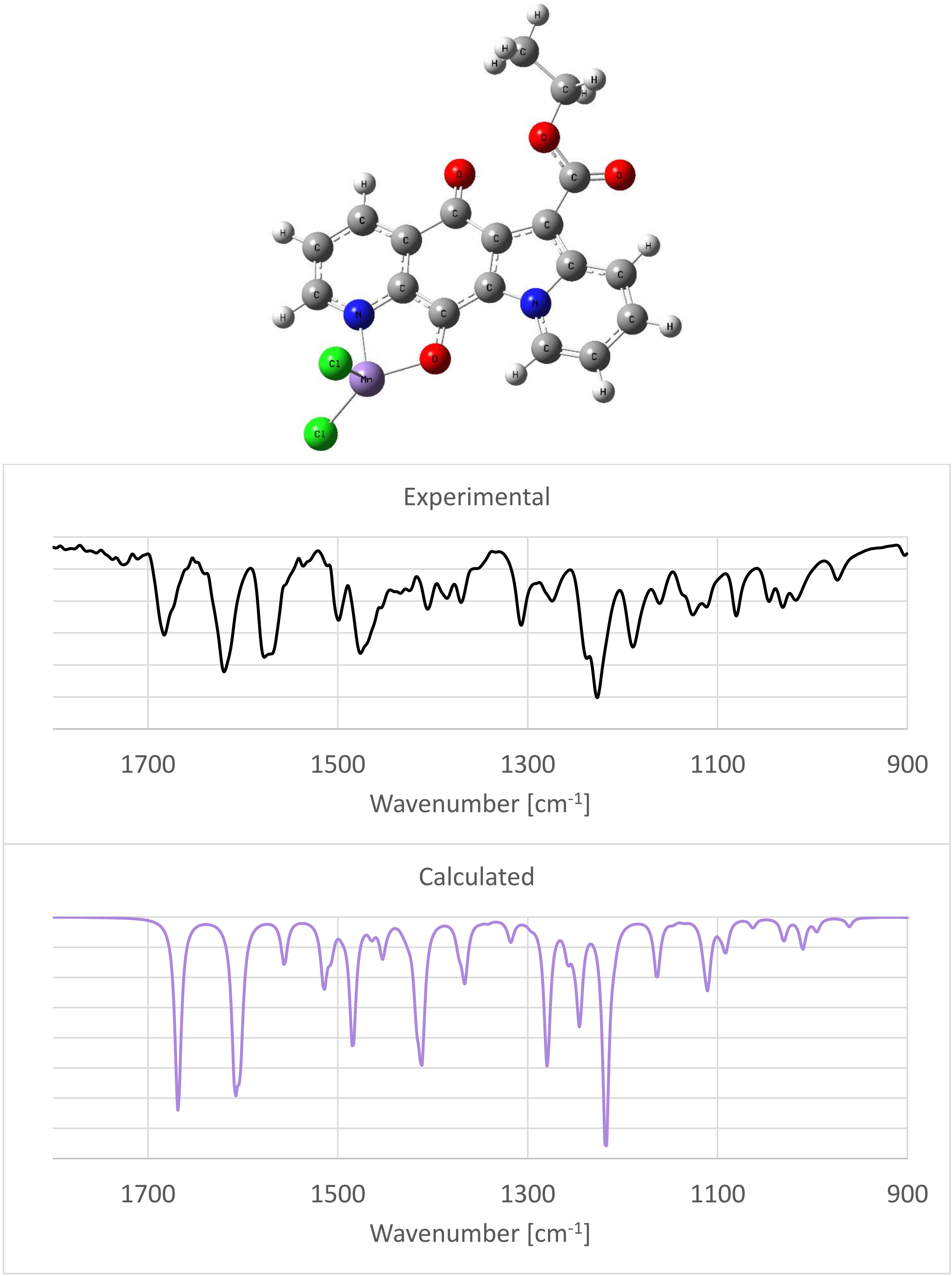

Figure S8. FT-IR spectrum and DFT-calculated spectrum at the B3LYP-GD3BJ/ 6-311+G(d,p) level of theory, with the energy-minimized structure of manganese complex **10**.

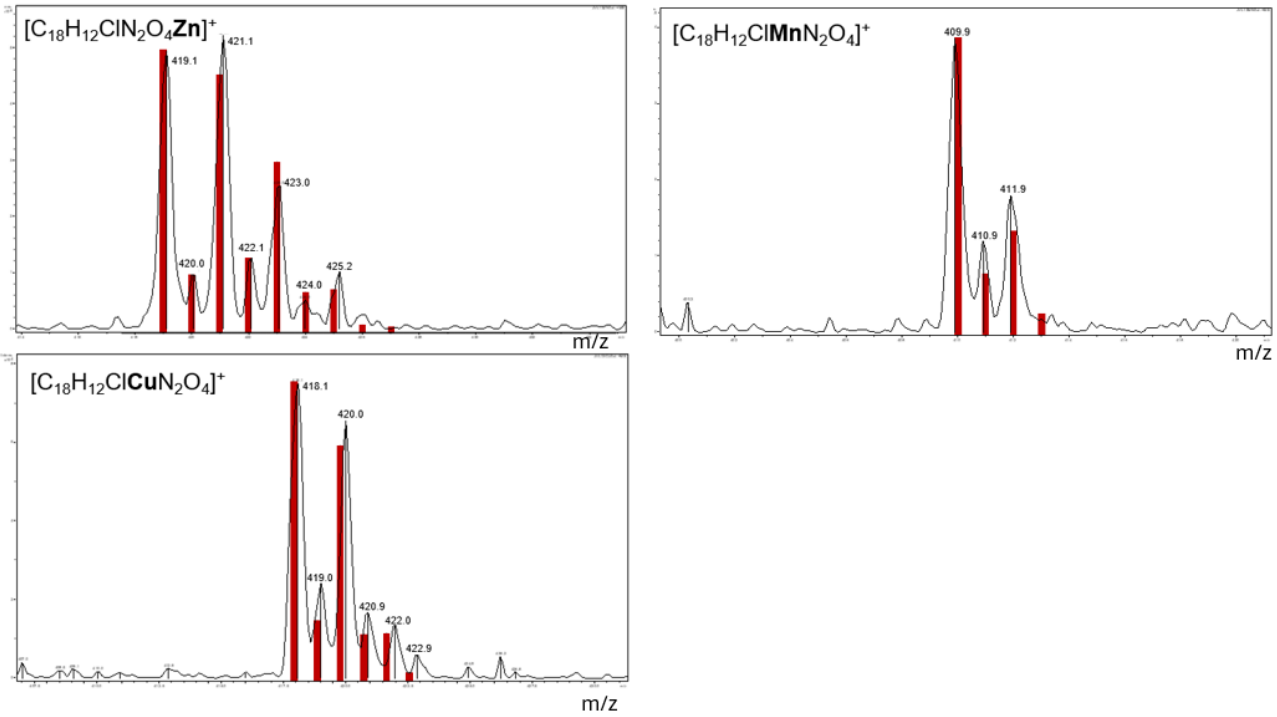

Figure S9. ESI(+) MS analysis of the complexes **8–10**: experimental (black) and simulated (red) isotopic clusters of the $[M\text{-}Cl]^+$ ions, where M= complexes of the indicated metal ions.
